# Intensive follow-up for women with breast cancer: review of clinical, economic and patient’s preference domains through evidence to decision framework

**DOI:** 10.1186/s12955-017-0779-5

**Published:** 2017-10-19

**Authors:** Alessandra Lafranconi, Liisa Pylkkänen, Silvia Deandrea, Anke Bramesfeld, Donata Lerda, Luciana Neamțiu, Zuleika Saz-Parkinson, Margarita Posso, David Rigau, Ivan Sola, Pablo Alonso-Coello, Maria José Martinez-Zapata

**Affiliations:** 1 CESP, School of Medicine, Università degli Studi Milano Bicocca, Monza, MB Italy; 2 Department of International Health, FHML, CAPHRI, Maastricht University, Maastricht, Netherlands; 3 Cancer Society of Finland, Helsinki, Finland; 4 European Commission, Directorate General Joint Research Centre (JRC), Directorate F - Health, Consumers and Reference Materials, Ispra, VA Italy; 5 Iberoamerican Cochrane Centre - Service of Clinical Epidemiology and Public Health, Biomedical Research Institute Sant Pau (IIB Sant Pau), Barcelona, Spain

**Keywords:** Breast cancer, Follow-up, Recommendation, EtD framework

## Abstract

**Background:**

Women treated for breast cancer are followed-up for monitoring of treatment effectiveness and for detecting recurrences at an early stage. The type of follow-up received may affect women’s reassurance and impact on their quality of life. Anxiety and depression among women with breast cancer has been described, but little is known about how the intensity of the follow-up can affect women’s psychological status. This study was undertaken to evaluate the effects of intensive vs. less-intensive follow-up on different health outcomes, to determine what are women’s preferences and values regarding the follow-up received, and also assess the costs of these different types of follow-up.

**Methods:**

A systematic review following standard Cochrane Collaboration methods was carried out to assess the efficacy of intensive follow-up versus non-intensive follow-up in breast cancer patients. Two additional reviews on women’s preferences and economic evidence were also carried out. The search was performed up to January 2016 in: MEDLINE, EMBASE, PDQ, McMaster Health Systems Evidence, CENTRAL, and NHS EED (through The Cochrane Library). The quality of evidence was assessed by GRADE (for quantitative studies) and CerQUAL (for qualitative studies). Several outcomes including mortality, breast cancer recurrences, quality of life, and patient satisfaction were evaluated.

**Results:**

Six randomised trials (corresponding to 3534 women) were included for the evaluation of health outcomes; three studies were included for women’s values and preferences and four for an economic assessment. There is moderate certainty of evidence showing that intensive follow-up, including more frequent diagnostic tests or visits, does not have effects on 5- or 10-year overall mortality and recurrences in women with breast cancer, compared with less intensive follow-up. Regarding women’s preferences and values, there was important variability among studies and within studies (low confidence due to risk of bias and inconsistency). Furthermore, intensive follow-up, as opposed to less intensive follow-up, is not likely to be cost-effective.

**Conclusions:**

Less intensive follow-up appears to be justified and can be recommended over intensive follow-up. Resources could thus be mobilised to other aspects of breast cancer care, or other areas of healthcare.

**Electronic supplementary material:**

The online version of this article (10.1186/s12955-017-0779-5) contains supplementary material, which is available to authorized users.

## Background

Breast cancer is the most frequently diagnosed cancer and among the leading causes of cancer death among females [1–3]. Due to significant improvements in screening, early diagnosis, and treatment in the recent decades, breast cancer mortality has decreased worldwide [4–6]. This leads to a situation where the total number of prevalent breast cancer cases is increasing, and therefore a growing number of women needing follow-up care. Worldwide and European estimates of women with a diagnosis of breast cancer occurring in the last 5 years correspond to 6.2 and 1.8 million, respectively [7].

Women treated for breast cancer are followed-up for monitoring treatment effectiveness and complications, and for detecting recurrences at an early stage or new primary contralateral breast cancer. Follow-up includes clinical and test examinations such as routine haematological and liver function tests, tumour markers, chest X-ray, mammography and bone and liver scans [8]. The diversity in frequency and in the type of examination results in many different follow-up practices, the intensity of which can be defined by the frequency of clinical visits and/or physical examinations (e.g. intensive, standard, patient-initiated or low intensity). There is also evidence of variability [9] in the way follow-up is implemented in clinical practice.

Despite doubts that intensive follow-up care can improve survival in these patients, intensive follow-up is quite common in clinical practice and represents a significant workload for radiotherapy, surgical and oncologic departments [10].

For a long time, the scientific community has focused on the relationship between the type of follow-up (i.e. intensity) and health outcomes, such as long-term mortality and morbidity, but also quality of life. A recent Cochrane review that analysed randomised control trials with almost 20 years of follow-up gave the following suggestion: "*follow-up programs based on regular physical examinations and yearly mammography alone are as effective as more intensive approaches based on regular performance of laboratory and instrumental tests in terms of timeliness of recurrence detection, overall survival and quality of life*" [11].

Despite the importance of health outcomes in terms of mortality and morbidity, it is also important to take into account the women’s perspective, including psychosomatic symptoms and diseases, which could be manifested as preference towards one or another type of follow-up scheme [12]. Similarly, economic evidence in healthcare is becoming increasingly important, not only in the form of cost-effectiveness or cost-utility analyses, which are the most common mechanism for generating economic evidence in decision making, but also in the form of cost-minimisation, cost-consequences or cost-benefit analyses or total budget impact estimates [13]. This is another key point to be considered in recommending a certain type of follow-up protocol.

Given that all these aspects should be considered together to make decisions in healthcare, there is an urgent need to use up-to-date and user-friendly evidence-presentation formats, in order to improve the communication of evidence-based healthcare recommendations, addressing communication needs of guideline users and decision-makers [14]. The Grading of Recommendations Assessment, Development, and Evaluation (GRADE) methodology [15–17] in combination with Evidence to Decision (EtD) Frameworks provides an assessment and a summary of alternative strategies on three key elements: patient-important outcomes, patients’ values and preferences, and economic evidence. Information regarding acceptability and feasibility of the analysed strategies, and their impact on health equity is also included [15, 18].

The aim of the present paper is to evaluate the available research evidence on the clinical question about whether intensive follow-up should be provided for breast cancer patients treated with curative intent. The evidence is assessed and summarised according to GRADE and the EtD framework; the recommendations made in this manuscript are based on the authors judgements and should only be considered as the authors’ recommendations and not as recommendations made by a guideline panel. Nevertheless, they are useful to facilitate the further decision-making process carried out by guideline panels in charge of issuing clinical recommendations.

## Methods

### Systematic review on the evidence of effects of intensive follow-up on breast cancer outcomes

The research question was addressed by means of a systematic review of the literature on the evidence of health outcomes related to the alternative strategies – intensive and less intensive follow-up. An operational definition was used for intensive follow-up, where intensive was defined in comparison with a less intensive follow-up schedule or a patient-initiated approach. The review protocol is available upon request. Standard Cochrane Collaboration methods were followed [19]. For the evaluation of the importance of the outcomes and for the assessment of the quality of evidence, the GRADE system was used.

**Research question**: the clinical question was structured following the PICO (Patient, Intervention, Comparison, Outcomes) format:

- Population: breast cancer patients, treated with curative intent;
- Intervention: intensive follow-up schedule;
- Comparison: non-intensive follow-up;
- Outcomes: 5- and 10-year mortality due to breast cancer; 5 and 10-year breast cancer recurrences (loco-regional and distant separately); 5- and 10-year breast cancer specific survival; quality of life at 2 and 5 years after diagnosis; women’s satisfaction with follow-up (measured by reassurance of women with the intensive follow-up and convenience by the women of intensive follow-up).

Critical outcomes included mortality due to breast cancer, breast cancer recurrences and breast cancer specific survival. Quality of life and satisfaction were considered important outcomes.

### Inclusion and exclusion criteria

Following the WHO Handbook for Guidelines Development [20] as guidance, existing relevant systematic reviews of observational and experimental evidence were included as a source of individual studies; additional individual studies were searched, to update the body of evidence. Temporal or language restrictions were not applied. Studies in which the effects of follow-up intensity were not assessed, or when the outcomes were out of the scope of the clinical question, were excluded.

### Search strategy

Systematic reviews were identified by introducing a combination of controlled vocabulary and search terms (e.g., follow-up, breast neoplasms, mortality, recurrences, quality of life, satisfaction, cost, healthcare resources, survival) in The Cochrane Database of Systematic Reviews (2015, issue 11), The Database of Abstracts of Reviews of Effects (DARE), and PubMed limiting the search to the subset “systematic [sb]”.

Original studies were searched in MEDLINE (through PubMed; from 1946 to January 2016), EMBASE (through Ovid; from 1980 to November 2015), PDQ, McMaster Health Systems Evidence, CENTRAL, and NHS EED (through The Cochrane Library; January 2016).

**Fig. 1:**
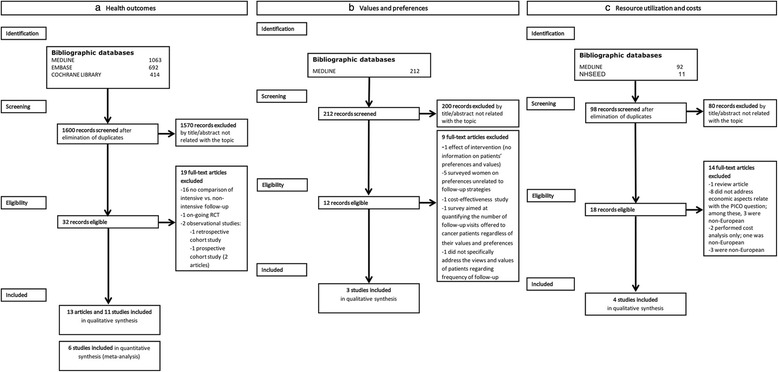
PRISMA flowcharts. Legend: Flowcharts representing the selection of studies for health outcomes (**a**), values and preferences (**b**), and resource utilisation and costs (**c**)

The complete search algorithms designed for each database, the hits retrieved, and the reasons for exclusion are presented in Additional file 1 and Fig. 1a.

One reviewer screened the search results based on title and abstract. This process was subjected to a quality process, by reviewing 20% of the references by a second reviewer. Two reviewers independently confirmed eligibility, based on the full text of the relevant articles. In case of disagreement between reviewers the inclusion of studies was determined by consensus.

### Data extraction

Data extraction was conducted by one reviewer. As quality control, another reviewer went through 20% of the data for accuracy.

### Risk of bias

The assessment was carried out by one reviewer. As quality control, another reviewer went through 20% of the data for accuracy. For each study, the risk of bias was rated for each domain as low, high or unclear risk of bias.

### Effect measures

Odds ratios (OR), risk ratios (RR) and hazard ratios (HR) were extracted, with their 95% confidence intervals (CI). If available, only adjusted effect measures were collected. Data from any estimation of effect provided (percentages, means, medians) were also collected.

### Quality of the evidence evaluation

The quality of evidence per outcome was rated from high to very low considering the standard GRADE domains: risk of bias, imprecision, inconsistency, indirectness and publication bias [21, 22].

### Data analysis

A pooled analysis was conducted applying the inverse-variance method under the random-effects model [23]; the analysis was performed through the Software Review Manager v. 5.3. Heterogeneity was assessed using the I^2^ statistic.

The report of results of the meta-analysis adhered to the guidelines articulated in the Preferred Reporting Items for Systematic Reviews and Meta-Analyses (PRISMA) statement [24].

### Review on women’s values and preferences

A review about women’s values and preferences for intensive follow-up versus non-intensive follow-up after breast cancer treatment was undertaken.

### Inclusion and exclusion criteria

In a first stage, after conducting the systematic search of the literature, the screening of references was carried out, initially prioritising the identification of systematic reviews. In a second stage, individual studies were retrieved (e.g., qualitative studies, surveys, utility elicitation studies). Studies in English, French, German, Greek, and Spanish, carried out in the Organisation for Economic Co-operation and Development (OECD) Region, were included. Included studies were:

- examining women’s preferences for follow-up strategies after breast cancer treatment;
- evaluating how women value the main outcomes associated with follow-up strategies after breast cancer treatment;
- examining the choices women make when informed about the desirable and undesirable effects associated with follow-up strategies after breast cancer treatment.

Studies assessing only women’s knowledge, views, perceptions, attitudes and expectations regarding follow-up strategies after breast cancer treatment were excluded; similarly, studies assessing barriers to follow-up strategies after breast cancer treatment were not included.

### Search strategy

A search strategy was designed to identify relevant studies in MEDLINE (accessed through Ovid). For systematic reviews, there were no time restrictions. For primary studies, only studies published after 2006 were included. The complete search strategy can be found in Additional file 2.

One reviewer screened the search results based on the title and abstract. Two reviewers independently confirmed eligibility based on the full text of the relevant articles. In case of disagreement between researchers, the inclusion of studies was decided by consensus (Fig. 1b).

### Data extraction

One reviewer extracted the main characteristics of the included studies and their findings in a tabulated format. A second reviewer checked the extracted data for accuracy.

### Quality of the evidence evaluation

The quality of evidence was rated with GRADE. In the case of qualitative research, the Confidence in the Evidence from Reviews of Qualitative research (CERQual) approach was used [25].

### Review on economic evidence

A review about the economic evidence for intensive follow-up versus non-intensive follow-up after breast cancer treatment was carried out.

### Inclusion and exclusion criteria

Screening of literature and study selection was done in a step–by- step approach. Firstly, the search focused on studies that addressed economic aspects directly related to the PICO question. Then, recent European cost-effectiveness or cost-utility analyses related to the PICO question were looked for. Only studies in English were included.

### Search strategy

Search strategies were designed to identify relevant studies in MEDLINE (through Ovid, January 2016) and in the NHS Economic Evaluation Database (through The Cochrane Library, January 2016). The complete search strategies are included in Additional file 3. Study design filters were applied to retrieve relevant studies. The selection process is presented in Fig. 1c.

### Data extraction

Main characteristics of included studies were described in a tabulated format, including the following data: author and publication year, country, type of economic analysis, perspective of the analysis, time horizon and discounting, relevant outcomes and costs included, sources of information (baseline outcomes, relative intervention effects, resource use and costs), Quality Adjusted Life Years (QALY), Incremental Cost Effectiveness Ratio (ICER), sensitivity analysis and conflict of interest.

### Quality of evidence

The quality of evidence for the resource requirements was rated according to GRADE [26]. The NICE methodology checklist for economic evaluations [27] was used to assess the risk of bias and decide whether to include the studies. Included studies were of low risk of bias and were considered applicable to the European context.

### Evidence to decision framework

To summarise the evidence, and in accordance to the GRADE methodology [28] and the interactive Evidence to Decision framework guidance [29], an EtD Framework was developed. The authors covered the role of the panel with respect to the EtD framework.

## Results

### Evidence of effects of intensive follow-up on breast cancer outcomes

Five systematic reviews were included for the evaluation of health outcomes [30–34]. These systematic reviews were used as a source to identify primary studies. Eight papers, referring to six randomised clinical trials for a total of 3534 randomised women [35–42], were retrieved and included. These studies are summarised in Table 1.

**Table 1:** Summary and short description of the six included randomised clinical trials

Study	Participants	Intervention	Comparator	Outcome	Risk of bias
Oltra 2007 RCT	Spain, hospital setting, 58 cases and 63 controls	Intensive follow-up: Outpatient appointments (following ASCO guidelines in frequency) had: anamnesis and physical examination, biochemistry, blood count, and the markers carcinoembryonic antigen (CEA) and CA15–3. Annual check-up included: mammography, hepatic echography, chest X-ray, and bone scan.	Standard follow up: Outpatient appointments (following ASCO guidelines in frequency) had anamnesis and physical examination; no complementary tests in absence of clinical symptoms. Annual check-up included mammography.	Cost-benefit evaluation (intensive vs. standard follow-up) in the early detection of breast cancer relapses.	- Random sequence generation: unclear - Allocation concealment: unclear - Blinding of participants and personnel: high risk - Blinding of outcome assessment: high risk - Incomplete outcome data: low risk - Selective reporting: low risk
Kokko 2003 Kokko 2005 RCT	Finland, hospital setting, 243 cases and 229 controls	Patient-initiated follow-up: Chest X-rays and other diagnostic tests taken only when clinically indicated. Moreover, patients were further randomised into: - outpatient appointments every 3 months (group A); - outpatient appointments every 6 months (group C).	Standard follow-up: Chest X-rays and other diagnostic tests taken routinely every 6 months. Moreover, patients were further randomised into: - outpatient appointments every 3 months (group B); - outpatient appointments every 6 months (group D).	Main study: recurrences, free disease survival, overall survival. Cost-benefit study: evaluation of the early detection of breast cancer relapses (appointments every 3 vs. 6 months, and routine vs. clinically-requested exams).	- Random sequence generation: unclear - Allocation concealment: unclear - Blinding of participants and personnel: high risk - Blinding of outcome assessment: high risk - Incomplete outcome data: low risk - Selective reporting: low risk
Brown 2002 Multicentre RCT	England, hospital setting (4 clinics), 31 cases and 30 controls	Patient-initiated follow-up: Patients received written information on the signs and symptoms of recurrence, and the invitation to contact the nurses by telephone in case of any problem. They did not attend routine clinic appointments. Annual check-up with mammography.	Standard follow-up: Outpatient appointments as standard clinic follow-up: anamnesis, physical examination, and possibility to ask questions. Annual check-up with mammography.	Quality of life. Psychological morbidity. Satisfaction with follow-up.	- Random sequence generation: low risk - Allocation concealment: unclear - Blinding of participants and personnel: high risk - Blinding of outcome assessment: high risk - Incomplete outcome data: low risk - Selective reporting: low risk
Gulliford 1997 Multicentre RCT	England, hospital setting (2 clinics), 97 cases and 96 controls	Patient-initiated follow-up: Outpatient visits only after mammography: yearly (lumpectomies done less than 5 years before; mastectomies performed less than 1 year before) or every other year (lumpectomies done more than 5 years before; mastectomies performed more than 1 year before). Patient-initiated phone contact in case of symptoms.	Standard follow-up: Outpatient visits according to conventional schedule: every 3 months if the surgery took place less than one year before; every four months if the surgery was between one and two years before; every six months if the surgery was between two and five years before; and annually if the surgery was more than five years before. Mammography as the other arm. Phone contact as the other arm.	Interim use of telephone and general practitioner. Satisfaction with allocation to follow-up.	- Random sequence generation: unclear - Allocation concealment: unclear - Blinding of participants and personnel: high risk - Blinding of outcome assessment: high risk - Incomplete outcome data: low risk - Selective reporting: low risk
Rosselli del Turco 1994 Palli 1999 Multicentre RCT	Italy, hospital setting (12 clinics), 622 cases and 621 controls	Intensive follow-up: Physical examination performed every 3 months in the first 2 years and every 6 months in the following 3 years; 2-view chest X-rays and bone scan performed every 6 months; mammography performed every year.	Standard follow-up: Physical examination performed every 3 months in the first 2 years and every 6 months in the following 3 years; mammography performed every year during the study (5 years). Other diagnostic tests performed only in presence of symptoms.	Overall survival. Relapse-free survival. Distant metastases. Death. Event-free survival (distant metastases or death).	- Random sequence generation: unclear - Allocation concealment: low risk - Blinding of participants and personnel: high risk - Blinding of outcome assessment: high risk - Incomplete outcome data: low risk - Selective reporting: low risk
GIVIO 1994 Multicentre RCT	Italy, hospital setting (26 clinics), 655 cases and 665 controls	Intensive follow-up: Physical exam every 3 months for 2 years and then every 6 months for 3 years; blood test every visit (ALP, gammaGT); chest X-rays every 6 months; annual radionuclide bone scan; annual liver echography; annual contralateral mammography.	Standard follow-up: Physical exam every 3 months for 2 years and then every 6 months for 3 years; annual contralateral mammography.	Mortality/overall survival. Recurrence (type of recurrence, time to detection of recurrence). Symptomatic status at diagnosis of metastases. Health-related quality of life.	- Random sequence generation: low risk - Allocation concealment: low risk - Blinding of participants and personnel: high risk - Blinding of outcome assessment: high risk - Incomplete outcome data: low risk - Selective reporting: low risk

The included studies had different definitions for intensive follow-up. In four studies, intensive follow-up referred to a greater number of diagnostic tests compared to regular follow-up [35, 36, 40, 42], while in two studies it referred to more frequent visits without modification in the number of diagnostic tests [37, 39]. Three studies compared an intensive versus a standard follow-up [35, 36, 42], while the other three compared a low-intensity patient-initiated versus a standard follow-up [37, 39, 40]. Five studies specified that patients (including the non-intensive follow-up group) underwent an annual mammography [35–38, 42]. No studies provided information about specific breast cancer mortality or survival. Among all studies, only the one carried out by the GIVIO group [35] reported the expected 5-year relative mortality reduction used for the calculation of sample size, i.e. 20% reduction; this threshold may be considered as the clinically significant mortality reduction expected.

**Fig. 2:**
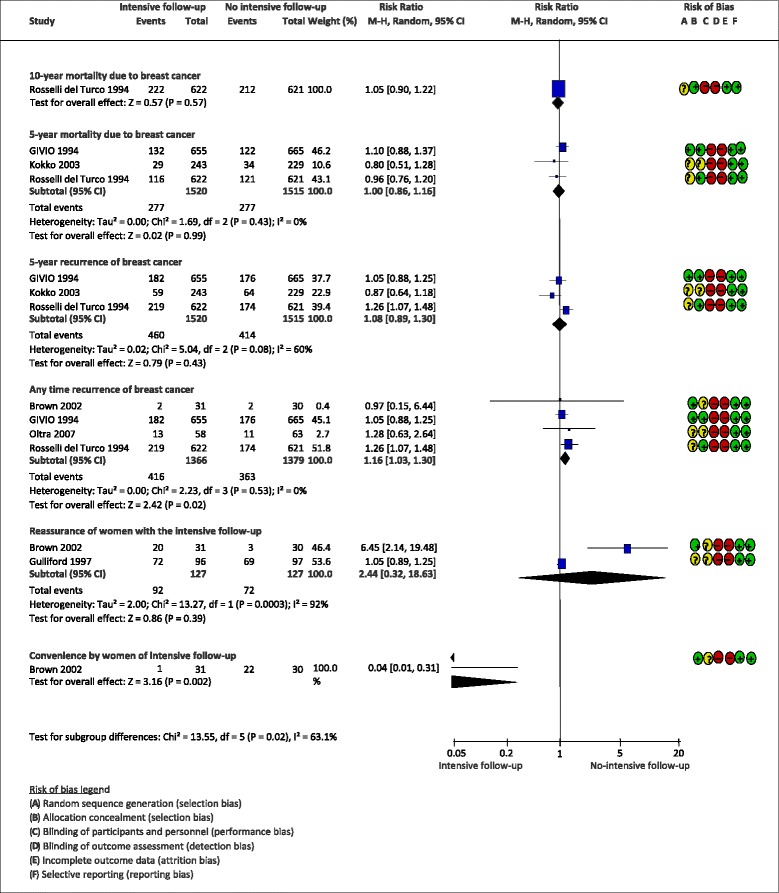
Estimates of effect of intensive vs. standard follow-up on breast cancer outcomes

Results and pooled analysis are provided when possible. Quantitative estimates are available only for the following outcomes, presented in Fig. 2:

**10-year overall mortality**: one trial [38], that compared intensive vs. standard follow-up in 1243 women, found a RR = 1.05 (95%CI: 0.90 to 1.22). The quality of evidence was high because non-blinding was not considered a cause of risk of bias for this outcome.

**5-year overall mortality**: three trials [35, 36, 40], on 3035 patients, that compared intensive vs. standard follow-up found a RR 1.00 (95%CI: 0.86 to 1.16; I^2^ = 0%). The 5-year mortality RR lower 95%CI did not reach the expected value for clinical significance either (vs. an expected 20% mortality reduction, as per GIVIO group outcome). The quality of evidence was high because non-blinding was not considered a cause of risk of bias for this outcome.

**5-year breast cancer recurrences (any loco-regional and distant)**: three trials [35, 36, 40], on 3035 patients, that compared intensive and standard follow-up, resulted in a RR = 1.08 (95%CI: 0.89 to 1.30; I^2^ = 60%). The quality of evidence was moderate because it was downgraded for risk of bias (the outcome assessment was not blinded). From the clinical point of view, when the patient is diagnosed with recurrence, there will be an initiation of new treatments or change in the treatment, so we considered that non-blinding is not an issue in this case.

**Breast cancer recurrences at any time**: five trials [35, 36, 39, 40, 42], on 3217 women, found a RR = 1.10 (95%CI: 0.95 to 1.27; I^2^ = 23%, when comparing intensive vs. standard follow-up. The quality of evidence was moderate because it was downgraded for risk of bias (the outcome assessment was not blinded). As discussed above, non-blinding was not considered as an issue.

**Satisfaction of women with the type of follow-up**: satisfaction was measured as *reassurance* (capacity of the type of follow-up to clear patients’ doubts or fears) in two studies [37, 39] on 245 patients, and as *convenience* (suitability of the follow-up to the woman’s life circumstances) in one study [39] on 61 women. The results on reassurance were in favour of intensive follow-up (RR 1.28, 95%CI: 1.07 to 1.54; I^2^ = 90%). The overall quality of evidence was very low due to risk of bias (the studies were not blinded) and imprecision (the number of events is small). The results on convenience favoured non-intensive follow-up (RR 0.04, 95%CI: 0.01 to 0.31). The overall quality of evidence was low because evidence needed to be downgraded for risk of bias (the outcome assessment was not blinded) and imprecision (the number of events is very small).

For each studied outcome, the evidence profile is reported in Table 2.

**Table 2:** Evidence profiles for selected health outcomes related to intensive vs. standard follow-up in breast cancer patients

Quality assessment							No. of patients		Effect		Quality	Importance
No. of studies	Study design	Risk of bias	Inconsistency	Indirectness	Imprecision	Other considerations	Intensive follow-up	Non-intensive follow-up	Relative (95% CI)	Absolute (95% CI)		
10-year overall mortality in women with breast cancer												
1	RCT	not serious	not serious	not serious	not serious	none	222/622 (35.7%)	212/621 (34.1%)	**RR 1.05**	**17 more per 1000**	⨁⨁⨁⨁	CRITICAL
Palli 1999									(0.90 to 1.22)	(from 34 fewer to 75 more)	HIGH	
5-year overall mortality in women with breast cancer												
3	RCT	not serious	not serious	not serious	not serious	none	277/1520 (18.2%)	277/1515 (18.3%)	**RR 1.00**	**0 fewer per 1000**	⨁⨁⨁⨁	CRITICAL
Rosselli del Turco 1994									(0.86 to 1.16)	(from 26 fewer to 29 more)	HIGH	
GIVIO 1994												
Kokko 2003												
5-year recurrence of breast cancer												
3	RCT	serious^1^	not serious	not serious	not serious	none	460/1520 (30.3%)	414/1515 (27.3%)	**RR 1.08**	**22 more per 1000**	⨁⨁⨁ ◯	CRITICAL
Rosselli del Turco 1994									(0.89 to 1.30)	(from 30 fewer to 82 more)	MODERATE	
GIVIO 1994												
Kokko 2003												
Any time recurrence of breast cancer (follow up: range 1 to 5 years)												
5	RCT	serious^1^	not serious	not serious	not serious	none	475/1609 (29.5%)	427/1608 (26.6%)	**RR 1.10**	**27 more per 1000**	⨁⨁⨁ ◯	CRITICAL
Rosselli del Turco 1994									(0.95 to 1.27)	(from 13 fewer to 72 more)	MODERATE	
GIVIO 1994												
Brown 2002												
Kokko 2003												
Oltra 2007												
Satisfaction of women with the type of follow-up - Reassurance (follow-up: range 1 to 3 years)												
2	RCT	serious^1^	very serious^2^	not serious	not serious	none	92/127 (72.4%)	72/127 (56.7%)	**RR 1.28**	**159 more per 1000**	⨁ ◯◯◯	IMPORTANT
Brown 2002									(1.07 to 1.54)	(from 40 more to 306 more)	VERY LOW	
Gulliford 1997												
Satisfaction of women with the type of follow-up - Convenience (follow-up: mean 1 year)												
1	RCT	serious^1^	not serious	not serious	not serious	none	1/31 (3.2%)	22/30 (73.3%)	RR 0.04	704 fewer per 1000	⨁ ⨁◯◯	IMPORTANT
Brown 2002									(0.01 to 0.31)	(from 506 fewer to 726 fewer)	LOW	

Legend: Abbreviations: RCT: Randomised clinical trial; CI: Confidence interval; RR: Risk Ratio; HR: Hazard Ratio. Notes: ^1^The quality of evidence was downgraded because studies were not blinded; ^2^The quality of evidence was downgraded due to important heterogeneity

### Women’s values and preferences

For women’s values and preferences, three European studies were included [37, 43, 44] (Table 3).

**Table 3:** Summary and short description of the three included studies on women’s preferences and values

Study	Participants	Intervention	Results	Risk of bias
Gulliford 1997 RCT	96 patients in conventional follow-up and 95 patients in non-conventional follow-up	Comparison of conventional follow-up (clinic visits, every three, four, six or 12 months, based on the time distance from the surgery) with non-conventional follow-up (clinical visits every 12 or 24 months). Mammography in both groups every 12 or 24 months.	Twice as many patients in both groups expressed a preference for reducing rather than increasing follow-up visits. No increased use of local practitioner services or telephone triage was recorded in the group with less-intensive follow-up.	Low risk of bias
Stemmler 2008 Questionnaire in the context of a surveillance study	801 (30.1%) of 2658 eligible patients	Survey aimed to evaluate patients’ views on surveillance after breast cancer.	The majority of women confirmed the need for surveillance (95%), and 47.8% of the patients in the self-help group answered that there was a need for more intensive diagnostic effort during follow-up. The main expectation from an intensified follow-up was the increased sense of security (80%).	High risk of bias
Kimman 2010 Multicentre discrete-choice experiment survey	5 hospitals, 331 (59%) of 557 eligible patients	Survey aimed to assess: - preferred professional/s involved in follow-up; - preferred type of follow-up (in person vs telephone); - preferred follow-up schedule	The most preferred person to perform follow-up was the medical specialist, but a combination of the medical specialist and breast care nurse was also acceptable to patients. Face-to-face contact was strongly preferred over telephone contact. Follow-up visits every three months were preferred over visits every four, six, or 12 months.	Moderate risk of bias

Gulliford et al. [37] compared experiences of 193 patients with breast cancer, randomised into a group with a conventional schedule of clinic visits, and a group of less intensive follow-up. Both cohorts received identical mammography and were invited to call for immediate appointments if they detected symptoms. Stemmler et al. [43] conducted a surveillance study in a population of women with breast cancer; among the respondents, most (59%) belonged to an organised self-help group. Kimman et al. [44] conducted a multicentre discrete-choice experiment survey to measure the strength of preferences for several characteristics of breast cancer follow-up. The results of these three studies were inconsistent: in the first study, women appeared to prefer non-intensive follow-up schedules, while in the other two the preferences favoured intensive schedules. However, important variability was present among studies and within studies. There was low confidence in the evidence due to risk of bias and inconsistency.

The results of the review indicated that most of the regularly scheduled follow-up visits used further extensive laboratory and imaging procedures exceeding the quantity of examinations recommended in most of the current follow-up guidelines.

### Economic evidence

Four studies [41, 42, 45, 46] assessed resources used, costs and cost-effectiveness of intensive follow-up strategies.

Robertson et al. [46] conducted a cost-utility analysis in the UK and provided estimated costs (in 2008 value) for different mammographic surveillance regimens in women after breast cancer surgery. By assuming the cost of a mammography and of a clinical follow-up visit to be 71 and 110 €, respectively, in a cohort of 10,000 UK women with a mean age of 57, total costs varied from 3.27 million € (mammographic surveillance every 2 years) to 16.8 million € (yearly mammographic and clinical follow-up) for a 10-year surveillance period. The study used a Markov model and found that the most cost-effective strategy was surveillance with mammography alone, provided every 12 months since the incremental cost-effectiveness ratio (ICER) for this strategy compared to no surveillance was € 6051 per QALY gained.

A cost-utility evaluation conducted in The Netherlands [45] analysed data (costs in 2008 value) on 299 patients randomised into four groups: (1) hospital follow-up; (2) nurse-led telephone follow-up; (3) hospital follow-up plus a short educational group programme (EGP); and (4) nurse-led telephone follow-up plus EGP. Hospital follow-up plus EGP had an ICER of 236 € per QALY compared to the next best alternative nurse-led telephone follow-up plus EGP. The other two strategies were dominated (higher costs and fewer QALYs). The authors concluded that nurse-led telephone follow-up combined with a short EGP could be a cost-effective option. However, they did not estimate the ICER of this strategy compared to standard follow-up. Furthermore, the time horizon of the study (one year) was clearly insufficient to evaluate the cost-effectiveness of compared alternatives.

The study of Oltra et al. [42] found that an intensive follow-up characterised by multiple laboratory and imaging tests triples average costs of the standard clinical follow-up without differences in early detection of relapses during the three years of follow-up. The study of Kokko et al. [41] found that the most expensive strategy doubled the costs of the cheapest one without important differences in breast cancer recurrences among them.

The quality of the evidence on economic evidence was moderate due to indirectness. The study [56] considered in the evidence to decision framework was conducted in the UK, and the results may not be applicable to other European countries.

The EtD framework was applied to conclude the assessment. The research question is summarised in Table 4, while Table 5 represents the assessment, carried out in its 12 domains: among others, the certainty of evidence (e.g., no statistically significant differences in mortality) between different types of follow-up), the important uncertainty and variability in women’s values, and the cost-effectiveness of the intervention (which favours non-intensive schedules) are crucial elements in drawing conclusions.

**Table 4:** Summary of the research question

Should women be followed intensively after breast cancer treatment?			
Problem:	Women treated for breast cancer are followed-up for monitoring treatment effectiveness and for detecting recurrences at an early stage, but the frequency of follow-up is under discussion.	Background:	Women treated for breast cancer are followed up for monitoring treatment effectiveness and for detecting recurrences at an early stage. Follow-up includes clinical and test examinations like routine haematological and liver function tests, tumour markers, chest X-ray, mammography, bone and liver scans. There is variability in the frequency of medical visits and the tests performed.
Option:	Intensive follow-up.		
Comparison:	Non-intensive follow-up.		
Main outcomes:	1. 10-year mortality due to breast cancer. 2. 5-year mortality due to breast cancer. 3. 5 (or 10)-year breast cancer specific survival. 4. 10-year breast cancer recurrences (loco-regional and distant separately). 5. 5-year breast cancer recurrences (logo-regional and distant separately). 6. Quality of life of breast cancer patients 2 (or 5) years after diagnosis. 7. Patient satisfaction with follow-up.		
Setting:	Breast cancer centres/other healthcare services.		
Perspective:	Population.		

Legend: this table represents the first part of the Evidence to Decision framework

**Table 5:** Summary of the assessment on the research question

Domain	Judgement	Research evidence	Additional considerations
Problem	Is the problem a priority?	With over 458,000 new cases and 131,000 deaths per year, breast cancer is one of the main killers in Europe, and its diagnosis, treatment and follow-up represent major public health priorities. Despite the doubts that intensive follow-up care could improve survival in patients after breast cancer, intensive follow-up is quite common in clinical practice and represents a significant workload for radiotherapy, surgical and oncologic departments (Loprinzi 1994), and it is also costly.	
	○ No		
	○ Probably no		
	● Probably yes		
	○ Yes		
	○ Varies		
	○ Don’t know		
Desirable effects	How substantial are the desirable anticipated effects?	The evidence showed uncertain differences in overall mortality at 5 and 10-year follow-up (high quality evidence), and uncertain differences in recurrences at 5 years of follow-up (moderate quality evidence). The evidence showed significant differences in reassurance of women in favour of intensive follow-up (very low quality evidence), and convenience in favour of non-intensive follow-up (low quality evidence). There was missing research evidence in respect to the outcomes: 5 and 10-year breast cancer specific survival, 10-year breast cancer recurrences and quality of life of breast cancer patients 2 or 5 years after diagnosis.	
	● Trivial		
	○ Small		
	○ Moderate		
	○ Large		
	○ Varies		
	○ Don’t know		
Undesirable Effects	How substantial are the undesirable anticipated effects?		Undesirable health effects are related to mental health (stress for false positive, false reassurance for false negative).
	○ Large		
	○ Moderate		
	○ Small		
	● Trivial		
	○ Varies		
	○ Don’t know		
Certainty of evidence	What is the overall certainty of the evidence of effects?	The evidence on 5- and 10- year overall mortality was of high quality, and did not favour intensive versus standard follow-up. The evidence on 5-year cancer recurrences was of moderate quality, and there were uncertain differences between intensive and standard follo-up; similar conclusions apply to cancer recurrences at any time. The evidence of women satisfaction was of very low quality (reassurance domain) and of moderate quality (convenience domain). The evidence on values for women was of low quality (inconsistency among studies). The evidence on economic evaluations was of high quality, and favoured non-intensive follow-up.	
	○ Very low		
	○ Low		
	● Moderate		
	○ High		
	○ No included studies		
Values	Is there important uncertainty about or variability in how much people value the main outcomes?	Important variability was present among studies and within studies regarding women preferences for the intensity of follow-up (moderate confidence) (Gulliford 1997, Stemmler 2008, Kimman 2010).	
	○ Important uncertainty or variability		
	● Possibly important uncertainty or variability		
	○ Probably no important uncertainty or variability		
	○ No important uncertainty or variability		
	○ No known undesirable outcomes		
Balance of effects	Does the balance between desirable and undesirable effects favour the intervention or the comparison?	The evidence on health outcomes favours the comparison. The evidence on values for women is unclear: reassurance seems to favour the intervention (very low quality evidence), while convenience seems to favour the comparison (moderate quality evidence).The evidence on health outcomes favours the comparison.	
	○ Favours the comparison		
	● Probably favours the comparison		
	o Does not favour either the intervention or the comparison		
	○ Probably favours the intervention		
	○ Favours the intervention		
	○ Varies		
	○ Don’t know		
Resources required	How large are the resource requirements (costs)?	Moderate costs for the annual mammography option. Large costs could result for more intensive follow-up schedules that could include more than one mammography per year, clinical examinations, or MRI, or bone scans or others. Moderate costs for the annual mammography option.	
	○ Large costs		
	○ Moderate costs		
	○ Negligible costs and savings		
	○ Moderate savings		
	○ Large savings		
	● Varies		
	○ Don’t know		
Certainty of evidence of required resources	What is the certainty of the evidence of resource requirements (costs)?	Evidence comes from a good quality cost-utility analysis study from the UK (Robertson 2011).	
	○ Very low		
	○ Low		
	● Moderate		
	○ High		
	○ No included studies		
Cost effectiveness	Does the cost-effectiveness of the intervention favour the intervention or the comparison?	In the base-case scenario of a cost-utility analysis of different follow-up strategies carried out in the UK, the strategy with the highest net benefit, and most likely to be considered cost-effective, was surveillance mammography alone every 12 months at a societal willingness to pay for a quality-adjusted life year of either £20,000 or £30,000. The incremental cost-effectiveness ratio for surveillance mammography alone every 12 months compared with no surveillance was € 6051 (2008 value) (Robertson 2011).	Even though different countries use different cost per QALY thresholds for deciding which interventions will be funded by public health services, € 6051 is far below the threshold used in most European countries.
	● Favours the comparison		
	○ Probably favours the comparison		
	○ Does not favour either the intervention or the comparison		
	○ Probably favours the intervention		
	○ Favours the intervention		
	○ Varies		
	○ No included studies		
Equity	What would be the impact on health equity?		With less intensive follow-up strategies, resources could be mobilised to other aspects of breast cancer care or other areas of health care that could increase equity.
	○ Reduced		
	○ Probably reduced		
	○ Probably no impact		
	● Probably increased		
	○ Increased		
	○ Varies		
	○ Don’t know		
Acceptability	Is the intervention acceptable to key stakeholders?		Some patients, relatives and health professionals might find it unacceptable to reduce the number of visits and tests performed.
	○ No		
	○ Probably no		
	○ Probably yes		
	○ Yes		
	● Varies		
	○ Don’t know		
Feasibility	Is the intervention feasible to implement?		Settings with more intensive follow-up strategies will need to consider what is the impact of implementing less intensive strategies (e.g. relocate healthcare professionals or equipment).
	○ No		
	○ Probably no		
	● Probably yes		
	○ Yes		
	○ Varies		
	○ Don’t know		

Legend: This table is the second part of the Evidence to Decision framework

Finally, Table 6 reports the conclusions summarised by the authors, in the form of a suggestion to perform breast cancer follow-up once a year with a mammography visit, as opposed to other types of regimens.

**Table 6:** Authors’ conclusions and summary remarks on the research question

Should women be followed intensively after breast cancer treatment?					
Type of recommendation	Strong recommendation against the option	Conditional recommendation against the option	Conditional recommendation for either the option or the comparison	Conditional recommendation for the option	Strong recommendation for the option
	○	●	○	○	○
Recommendation	We suggest that women with breast cancer are followed-up once a year with a mammography (as opposed to other regimens) (provisional and conditional recommendation).				
Justification	There is moderate certainty of evidence that intensive follow-up compared with less intensive follow-up (more frequent diagnostic tests or visits) does not reduce 5–10-year overall mortality and recurrences in women with breast cancer. The cost of different regimens of follow-up is variable, with more intensive regimens being more expensive and cost-effectiveness favouring less intensive regimens. Resources could be mobilised to other aspects of breast cancer care, or other areas of healthcare, potentially increasing equity. This recommendation is provisional because of the uncertainty about the net benefit of the interventions. This recommendation is conditional because it might be different depending on the feasibility of the setting of the intensive follow-up policy.				
Subgroup considerations	Not applicable (no specific subgroup of women were considered).				
Implementation considerations	Women should be informed in detail at baseline about different types of follow-up and their related impacts, to increase their satisfaction and reassurance with a less intensive follow-up. Resources could be mobilised to other aspects of breast cancer care, or other areas of health care, potentially increasing equity.				
Monitoring and evaluation	Health outcomes related to less intensive follow-up should be periodically assessed (we suggest every 5 years).				
Research priorities	Patient-centred endpoints should be explored, and the relationship between follow-up intensity and technical and psychological support to continue endocrine treatment should be further studied. Similarly, organisational aspects related to the coordination of follow-up activities should be addressed.				

Legend: This table represents the third and last part of the Evidence to Decision framework

## Discussion

### Main findings

Our results showed that intensive follow-up, compared with less intensive follow-up including more frequent diagnostic test or visits, does not have beneficial effects on 5- or 10 -year overall mortality or recurrences in women with breast cancer. This finding was consistent between the studies included, and the quality of the evidence was moderate. Among the included studies, two randomised trials showed that intensive follow-up appeared to increase reassurance in patents (data on 250 women; RR 1.28, 95% CI from 1.07 to 1.54) [37, 39]. However, the quality of the studies was downgraded due to the inconsistency of studies. The cost of different regimens of follow-up is variable, with more intensive regimes being more expensive but without increases in health benefits; thus less intensive regimes are favoured. From one cost-utility analysis [46], an annual visit with mammography results in moderate costs, can be considered cost-effective compared to no surveillance, and is likely to be feasible.

### Our results in the context of previous results

The European Society of Medical Oncology (ESMO) Guidelines on breast cancer recommend regular visits every 3–4 months for the first 2 years after treatment (and gradually decreasing thereafter) in addition to an annual mammography [47]. American Cancer Society/American Society of Clinical Oncology Guidelines [8] also recommend detailed cancer-related history and physical examination every 3 to 6 months for the first 3 years after primary therapy (and thereafter decreasing) in addition to a yearly mammography. Their recommendations would fall under the definition of a “less intensive follow-up” that, in the majority of the studies included in our review, would include at least a clinical visit and mammography once a year. However, intensive follow-up is still also quite common in clinical practice [48–51] and represents a significant workload for radiotherapy, surgery and oncology professionals [10], in addition to being a costly process.

A recently published systematic review on the effects of breast cancer follow-up showed that standard approaches are as effective as intensive ones; moreover, no differences in quality of life were documented [1]. While considering the health outcomes, including mortality and recurrences, our results confirm the already reported results.

### Limitations and strengths

Many of the studies included in our review were carried out in previous decades, and their results might be slightly outdated, given the recent substantial changes in breast cancer care [52]; However, our study also took into account further perspectives, by including also women’s preferences and values, and economic aspects, as adopted in the GRADE approach. The reviews on women’s values and preferences and economic evidence were, however, limited to English and for the last ten years and Medline only, and results would have been more robust if such reviews were carried out with a broader scope. Moreover, the suggestion for less intensive follow-up was built by using the EtD: this is a new approach in the clinical oncology field, but has been previously used already in breast cancer screening [53], colon cancer screening [54], as well as in other contexts [55]. The EtD explicitly takes into account factors related, among others, to the quality of evidence, desirable and undesirable effects, values, resources and feasibility, that altogether constitute a comprehensive approach to a decision-making exercise. The suggestion reported in this paper was made by a multidisciplinary group of authors, but it should not be considered as a recommendation from a guideline panel.

### Implications for practice and research

The main expectation from an intensified follow-up from a women’s perspective was reassurance and increased sense of security. This finding raises the need to better inform women on the lack of evidence of effect of intensive follow-up on clinical outcomes of mortality and recurrences. However, it needs to be considered that the follow-up visit may also have additional aims than detection of recurrence, such as motivating women to continue endocrine treatment during the follow-up period, providing information about long-term adverse effects of treatment, and helping in their management, as well as providing psychosocial support [56]. These other aims of follow-up are very important in the light of the high prevalence of e.g., depression (varying from 9.4% to 66.1%), and anxiety (varying from 17.9% to 33.3%) among breast cancer survivors [12]. These additional aspects should not be neglected and they should be better explored while evaluating the effects of different follow-up strategies. Hence, further well-designed studies should be performed. There is a need to balance and prioritise these different outcomes, including also additional patient-centred endpoints described above, as well as including undesirable effects of more frequent investigations. Moreover, organisational aspects related to the coordination of follow-up activities (i.e. nurse-led and GP-led activities, etc.) are only analysed in few studies [57] and should be better explored, as they may impact on the acceptance of the protocol by women, healthcare providers, etc. as well as on costs and feasibility.

From the clinical point of view, annual mammography is well justified to detect potential new primary or local recurrences. On the contrary more intensive follow-up schedules including additional diagnostic tests, such as breast MRI, liver ultrasound or bone scans could result in large costs without sufficient evidence regarding their benefits or harms.

In summary, based on these findings, less intensive follow-up could be recommended, although the exact format of the follow-up visit would need to be further clarified, as the studies used quite different follow-up schedules and tests. The treatment of breast cancer has become increasingly individualised [58] as the risk of breast cancer recurrences is very variable and is related, among other variables, to genetic predisposition of individual women, breast cancer characteristics and its treatment. Therefore, also the follow-up should be individualised based on the risk estimates, and on women’s perceptions and values. A “one size fits all” approach may not be relevant.

## Conclusion

Based on the evaluation of clinical and economical outcomes carried out, a less intensive follow-up could be recommended. Patients should be provided with accurate information on the benefits (or lack of those) and harms of intensive follow-up. Resources could thus be mobilised to other aspects of breast cancer care, or other areas of healthcare, potentially increasing equity in society.

## Additional files

Additional file 1: Search strategy for the evidence of effects. (DOCX 100 kb)

Additional file 2: Search strategy for women’s values and preferences evidence. (DOCX 95 kb)

Additional file 3: Search strategy for economic evidence. (DOCX 97 kb)

## Abbreviations

CERQual: Confidence in the Evidence from Reviews of Qualitative research
CI: Confidence intervals
EtD: Evidence to Decision
GRADE: Grading of Recommendations Assessment, Development, and Evaluation
HR: Hazard ratios
ICER: Incremental Cost Effectiveness Ratio
OR: Odds ratios
PICO: Patient, Intervention, Comparison, Outcomes
QALY: Quality Adjusted Life Years
RR: Risk ratios
