# Supplementary material for: Intensive follow-up for women with breast cancer: review of clinical, economic and patient’s preference domains through evidence to decision framework
Source: Health Qual Life Outcomes. 2017 Oct 19;15:206. doi: 10.1186/s12955-017-0779-5 (PMC5649085; doi:10.1186/s12955-017-0779-5)
Supplement: Supplementary file 1 — Search strategy for the evidence of effects. (DOCX 100 kb) [file 12955_2017_779_MOESM1_ESM.docx]

**Search strategy for the evidence of effects**

| **Clinical questions:** Should women be intensively followed after breast cancer treatment? | |
| --- | --- |
| **Database and date** | **Search algorithm and hits retrieved** |
| **MEDLINE**  Ovid MEDLINE(R) In-Process & Other Non-Indexed Citations, Ovid MEDLINE(R) Daily and Ovid MEDLINE(R) <1946 to Present>  16.11.2015 | 1 exp breast cancer/ (240605)  2 breast cancer.ti,ab. (196755)  3 breast carcinoma*.ti,ab. (26388)  4 breast tumo?r*.ti,ab. (18451)  5 breast neoplasm*.ti,ab. (979)  6 (breast adj4 (cancer or carcinoma* or tumo?r or neoplasm*)).ti. (151632)  7 1 or 2 or 3 or 4 or 5 or 6 (292943)  8 (intensiv* adj5 follow up).ti,ab. (1363)  9 (intensity adj5 follow up).ti,ab. (523)  10 (high* adj5 follow up).ti,ab. (10014)  11 surveillance.ti,ab. (120682)  12 exp "Continuity of Patient Care"/ (16467)  13 (frequen* adj5 follow up).ti,ab. (3557)  14 (alternative* adj3 follow up).ti,ab. (263)  15 (routine adj3 follow up).ti,ab. (3002)  16 *Population Surveillance/ (17226)  17 8 or 9 or 10 or 11 or 12 or 13 or 14 or 15 or 16 (162497)  18 7 and 17 (4552)  19 limit 18 to systematic reviews (169)  20 Randomized Controlled Trial.pt. (416948)  21 Controlled Clinical Trial.pt. (92231)  22 randomi?ed.ab,ti. (435885)  23 placebo.ab,ti. (174646)  24 drug therapy.sh. (29209)  25 randomly.ab,ti. (245309)  26 trial.ab,ti. (417603)  27 groups.ab,ti. (1545812)  28 20 or 21 or 22 or 23 or 24 or 25 or 26 or 27 (2316068)  29 Animals/ (5651923)  30 Humans/ (14563333)  31 29 and 30 (1596541)  32 29 not 31 (4055382)  33 28 not 32 (1947151)  34 18 and 33 (959)  35 19 or 34 (1063) |
| **The Cochrane Library**  16.11.2015 | #1 MeSH descriptor: [Breast Neoplasms] explode all trees 9107  #2 breast cancer:ti,ab 18878  #3 breast carcinoma*:ti,ab 1495  #4 breast tumor*:ti,ab 3047  #5 breast tumour*:ti,ab 1037  #6 breast neoplasm*:ti,ab 94  #7 (breast near/4 (cancer or carcinoma* or tumor* or tumour* or neoplasm*)):ti 15135  #8 #1 or #2 or #3 or #4 or #5 or #6 or #7 20611  #9 MeSH descriptor: [Continuity of Patient Care] explode all trees 581  #10 (intensiv* near/5 follow up):ti,ab 206  #11 (intensity near/5 follow up):ti,ab 94  #12 (high* near/5 follow up):ti,ab 1159  #13 (frequen* near/5 follow up):ti,ab 356  #14 (alternative* near/5 follow up):ti,ab 70  #15 (routine near/5 follow up):ti,ab 308  #16 surveillance:ti,ab 2946  #17 #9 or #10 or #11 or #12 or #13 or #14 or #15 or #16 5555  #18 #8 and #17 241  Cochrane Database of Systematic Reviews: Issue 11 of 12, Nov 2015 13 hits  Database of Abstracts of Reviews of Effect: Issue 2 of 4, April 2015 12 hits  Cochrane Central Register of Controlled Trials: 10/12, Oct 2015 148 hits |
| **Embase**  Ovid <1980 to 2015 Week 45>  16.11.2015 | 1 exp breast cancer/ (321782)  2 breast cancer.ti,ab. (261925)  3 breast carcinoma*.ti,ab. (31867)  4 breast tumo?r*.ti,ab. (23452)  5 breast neoplasm*.ti,ab. (994)  6 (breast adj4 (cancer or carcinoma* or tumo?r or neoplasm*)).ti. (194939)  7 1 or 2 or 3 or 4 or 5 or 6 (392109)  8 (intensiv* adj5 follow up).ti,ab. (1985)  9 (intensity adj5 follow up).ti,ab. (764)  10 (high* adj5 follow up).ti,ab. (15323)  11 surveillance.ti. (37100)  12 (frequen* adj5 follow up).ti,ab. (5240)  13 (alternative* adj3 follow up).ti,ab. (378)  14 (routine adj3 follow up).ti,ab. (4527)  15 8 or 9 or 10 or 11 or 12 or 13 or 14 (64177)  16 7 and 15 (1701)  17 exp "systematic review"/ (98160)  18 meta analysis/ (101792)  19 systematic review*.ti,ab. (90291)  20 meta?nalys*.ti,ab. (5725)  21 meta analys*.ti,ab. (105641)  22 (MEDLINE and CENTRAL).ti,ab. (14280)  23 Cochrane.ti,ab. (50386)  24 17 or 18 or 19 or 20 or 21 or 22 (220502)  25 16 and 24 (46)  26 random*.tw. (1028124)  27 clinical trial*.mp. (1175795)  28 exp health care quality/ (2235729)  29 26 or 27 or 28 (3679074)  30 16 and 29 (678)  31 25 or 30 (692) |
