# Supplementary material for: Intensive follow-up for women with breast cancer: review of clinical, economic and patient’s preference domains through evidence to decision framework
Source: Health Qual Life Outcomes. 2017 Oct 19;15:206. doi: 10.1186/s12955-017-0779-5 (PMC5649085; doi:10.1186/s12955-017-0779-5)
Supplement: Supplementary file 2 — Search strategy for women’s values and preferences evidence. (DOCX 95 kb) [file 12955_2017_779_MOESM2_ESM.docx]

**Search strategy for women’s values and preferences evidence**

| **Clinical question:** Should women after breast cancer treatment be followed intensively? | |
| --- | --- |
| **Database and date** | **Search algorithm and hits retrieved** |
| **MEDLINE**  Ovid MEDLINE(R) In-Process & Other Non-Indexed Citations, Ovid MEDLINE(R) Daily and Ovid MEDLINE(R) <1946 to Present>  25.01.2016 | 1 *Choice Behavior/ (12557)  2 *Decision Making/ (30871)  3 *Attitude to Health/ (38156)  4 understanding.ti,ab. (541564)  5 perception*.ti,ab. (169061)  6 preference*.ti,ab. (104803)  7 attitude*.ti,ab. (107401)  8 expectation*.ti,ab. (61518)  9 (value or values).ti,ab. (1395718)  10 (view or views).ti,ab. (276234)  11 informed choice*.ti,ab. (1799)  12 informed decision*.ti,ab. (4644)  13 (women* adj5 decision*).ti,ab. (3113)  14 1 or 2 or 3 or 4 or 5 or 6 or 7 or 8 or 9 or 10 or 11 or 12 or 13 (2532841)  15 exp breast cancer/ (234290)  16 breast cancer.ti,ab. (191887)  17 (breast adj4 (cancer or carcinoma* or tumo?r or neoplasm*)).ti. (148244)  18 15 or 16 or 17 (281248)  19 (intensiv* adj5 follow up).ti. (156)  20 (intensity adj5 follow up).ti. (21)  21 (high* adj5 follow up).ti. (407)  22 surveillance.ti. (30094)  23 *"Continuity of Patient Care"/ (8118)  24 (frequen* adj5 follow up).ti. (71)  25 (alternative* adj3 follow up).ti. (24)  26 (routine adj3 follow up).ti. (230)  27 *Population Surveillance/ (16627)  28 19 or 20 or 21 or 22 or 23 or 24 or 25 or 26 or 27 (49162)  29 18 and 28 (819)  30 14 and 29 (147)  31 29 not 30 (672)  32 Qualitative Research/ (25013)  33 Focus Groups/ (19094)  34 qualitative.ti,ab. (141498)  35 interview*.ab. (227389)  36 focus group*.ti,ab. (26735)  37 purposive.ab. (4758)  38 theory.ab. (205334)  39 grounded theory.ab. (7025)  40 (mixed adj3 method*).ti,ab. (9300)  41 meta-ethnograph*.ti,ab. (227)  42 meta-synthe*.ti,ab. (341)  43 32 or 33 or 34 or 35 or 36 or 37 or 38 or 39 or 40 or 41 or 42 (541337)  44 31 and 43 (42)  45 29 not (30 or 44) (630)  46 exp Decision Support Techniques/ (64308)  47 (health adj3 utilit*).ti,ab. (2384)  48 gamble*.ti,ab. (3225)  49 prospect theor*.ti,ab. (170)  50 preference score*.ti,ab. (361)  51 (preference* adj5 elicitat*).ti,ab. (163)  52 health utilit*.ti,ab. (1301)  53 (utilit* adj3 (value* or score* or estimate*)).ti,ab. (3168)  54 (state adj5 utilit*).ti,ab. (752)  55 health state.ti,ab. (2909)  56 feeling thermometer*.ti,ab. (53)  57 best-worst scaling.ti,ab. (64)  58 standard gamble.ti,ab. (706)  59 time trade-off.ti,ab. (861)  60 TTO.ti,ab. (713)  61 probability trade-off.ti,ab. (15)  62 46 or 47 or 48 or 49 or 50 or 51 or 52 or 53 or 54 or 55 or 56 or 57 or 58 or 59 or 60 or 61 (74930)  63 45 and 62 (8)  64 29 not (30 or 44 or 63) (622)  65 barrier*.ti,ab. (187865)  66 64 and 65 (15) |
