# Supplementary material for: Intensive follow-up for women with breast cancer: review of clinical, economic and patient’s preference domains through evidence to decision framework
Source: Health Qual Life Outcomes. 2017 Oct 19;15:206. doi: 10.1186/s12955-017-0779-5 (PMC5649085; doi:10.1186/s12955-017-0779-5)
Supplement: Supplementary file 3 — Search strategy for economic evidence. (DOCX 97 kb) [file 12955_2017_779_MOESM3_ESM.docx]

**Search strategy for economic evidence**

| **NHS Economic Evaluation Database**  (The Cochrane Library)  12.01.2016 | #1 MeSH descriptor: [Breast Neoplasms] explode all trees 9121  #2 breast cancer:ti,ab 19314  #3 breast carcinoma*:ti,ab 1527  #4 breast tumor*:ti,ab 3152  #5 breast tumour*:ti,ab 1067  #6 breast neoplasm*:ti,ab 96  #7 (breast near/4 (cancer or carcinoma* or tumor* or tumour* or neoplasm*)):ti 15492  #8 #1 or #2 or #3 or #4 or #5 or #6 or #7 21061  #9 MeSH descriptor: [Continuity of Patient Care] explode all trees 583  #10 (intensiv* near/5 follow up):ti,ab 210  #11 (intensity near/5 follow up):ti,ab 98  #12 (high* near/5 follow up):ti,ab 1172  #13 (frequen* near/5 follow up):ti,ab 364  #14 (alternative* near/5 follow up):ti,ab 74  #15 (routine near/5 follow up):ti,ab 312  #16 surveillance:ti,ab 2991  #17 #9 or #10 or #11 or #12 or #13 or #14 or #15 or #16 5638  #18 #8 and #17 248  NHS Economic Evaluation Database : Issue 2 of 4, April 2015 11 hits |
| --- | --- |
| **MEDLINE**  Ovid MEDLINE(R) In-Process & Other Non-Indexed Citations, Ovid MEDLINE(R) Daily and Ovid MEDLINE(R) <1946 to Present>  12.01.2016 | 1 exp breast cancer/ (233873)  2 breast cancer.ti,ab. (191487)  3 breast carcinoma*.ti,ab. (26020)  4 breast tumo?r*.ti,ab. (17917)  5 breast neoplasm*.ti,ab. (962)  6 (breast adj4 (cancer or carcinoma* or tumo?r or neoplasm*)).ti. (147972)  7 1 or 2 or 3 or 4 or 5 or 6 (285757)  8 (intensiv* adj5 follow up).ti,ab. (1338)  9 (intensity adj5 follow up).ti,ab. (516)  10 (high* adj5 follow up).ti,ab. (9827)  11 surveillance.ti,ab. (117968)  12 exp "Continuity of Patient Care"/ (41001)  13 (frequen* adj5 follow up).ti,ab. (3504)  14 (alternative* adj3 follow up).ti,ab. (255)  15 (routine adj3 follow up).ti,ab. (2965)  16 *Population Surveillance/ (16592)  17 8 or 9 or 10 or 11 or 12 or 13 or 14 or 15 or 16 (183217)  18 7 and 17 (4435)  19 limit 18 to "economics (maximizes specificity)" (92) |
